# Exosomal long non-coding RNAs in lung cancer: A review

**DOI:** 10.1097/MD.0000000000038492

**Published:** 2024-12-20

**Authors:** Jingyuan Jiang, Fengwu Lin, Wenqi Wu, Zhe Zhang, Chen Zhang, Dongliang Qin, Zhenan Xu

**Affiliations:** a Department of Thoracic Surgery, China-Japan Union Hospital of Jilin University, Changchun, Jilin, China.

**Keywords:** exosomes, lncRNAs, lung cancer

## Abstract

Lung cancer is one of the most threatening malignancies among the different kinds of tumors. The incidence and mortality rate are increasing especially in male. Advances in diagnosis and treatment have been achieve in recent years. However, the lung tumor cells also developing chemo- and radio-resistance. Novel approaches and new treatments are stilled needed to develop for early diagnosis and treatment. Recently, long non-coding RNAs (lncRNAs) original exosomes were proved different expression in lung tumor, which mediate multiple biological processes and is responsible for tumor proliferation and metastasis. In this review, we focus on the emerging roles of both lncRNAs and exosomal lncRNAs in lung cancer and their roles on angiogenesis, metastasis, diagnosis, drug resistance, and immune regulation of lung cancer. Exosome lncRNAs were proved to serve as regulatory factors for gene expression, mediating intercellular communication, and participating in the occurrence and development of various diseases. In addition, exosomes lnc RNA has advantages on the early diagnosis of lung cancer, tumor cell metastasis, drug resistance, and immune regulation. Exosome lncRNAs an provide some unique ideas on how to improve the efficiency of diagnosis and treatment of lung cancer in the future.

## Introduction

Lung cancer is a malignant tumor with the fastest growth in incidence rate and mortality worldwide which largely threats the human life and health.^[1]^ Lung cancer can be divided into small cell lung cancer (15%) and non-small cell lung cancer (NSCLC) (85%) according to different tissue types.^[2]^ Although some progress in the early diagnosis and treatment of lung cancer are achieved in recent years, the current early diagnosis rate of lung cancer is only 15%, and most patients are diagnosed with advanced lung cancer when they had symptoms.^[3]^ The fundamental reason for this situation is the lack of effective early diagnosis methods. As the patients with lung cancer lacks symptoms in early stages, biopsies are an alternative diagnosis method. However, they are not recommended because of an invasive procedure. The vast majority of patients with mid to late stage have lost the opportunity for surgical treatment. Up to now, the exact mechanism of lung cancer metastasis is not yet fully understood, leading to a lack of effective method for early diagnosis and treatment. Therefore, exploring the pathogenesis of lung cancer from different perspectives has become an urgent problem to be solved.

Recent studies showed that exosomes are closely related to the occurrence and development of lung cancer.^[4–8]^ Exosomes can serve as intercellular messengers, affecting lung cancer cell proliferation, immunity, metastasis, drug resistance, and angiogenesis.^[9–13]^ Exosomes can also become ideal biomarkers for early diagnosis and treatment of lung cancer.^[14]^ In addition, studies reported that exosomes can serve as carriers to mediate drug delivery and be used for targeted treatment of lung cancer.^[15]^ Previous studies pointed that microRNAs (miRNAs) cargo in exosomes could be detected as biomarkers for lung cancer. However, long non-coding RNAs (lncRNAs) were also identified in exosomes, which seem to have more tissue specificity. Large numbers of lncRNAs are abnormally expressed in exosomes. In this study, we described lncRNAs specifically sorted in exosomes and summarized the roles of exosome lncRNAs in affecting the progression of lung tumor cells. We provide an overview of lncRNAs, exosomes, and their roles in angiogenesis, metastasis, diagnosis, drug resistance, and immune regulation of lung cancer.

## 2. The formation of exosomes

Electron microscopy showed that exosomes originate from endocytosis and undergo the formation of early and late endosomes. Then the cells endocytosis to form early endosomes, followed by invagination and protrusion of the envelope of the early endosomes. Early endosomes collected proteins and lipids from the cells cytoplasm, and formed multiple vesicles in the early endosomes, which turning into late endosomes.^[16]^ There are 2 metabolic pathways in late stage endosomes: one part is degraded by lysosomes after fusion with lysosomes. Some late endosomes migrate to the cell membrane, and fuses with the cell plasma membrane. Finally the cells released small vesicles into the extracellular matrix. These small vesicles were called exosomes.^[17]^ Exosomes have a lipid bilayer membrane structure, which is similar to cell membranes. Under the electron microscope, the exosomes can be observed to have a double concave disc or cup shape.^[18,19]^ The exosomes have a nanoscale particle structure and are relatively uniform in size. The diameter of exosomes generally ranges from 30 to 150 nm, and the density ranges from 1.13 to 1.21 g/cm^3^.

## 3. The extraction of exosomes

Exosomes are mainly extracted from cell culture supernatant or serum. The current methods for extracting exosomes include centrifugation, ultrafiltration, immunomagnetic bead, flow cytometry separation, and commercial exosomes extract kit. The centrifugation method involves gradually removing cell fragments and large microcapsules from the cell supernatant through different centrifugal forces, and ultracentrifugation to precipitate exosomes. The ultrafiltration method relies on a hollow fiber filter to remove interfering substances and then perform ultrafiltration to acquire exosomes. Research pointed that there is no significant difference in morphology and concentration between the exosomes extracted using these 2 methods. Compared to graded centrifugation, ultrafiltration is more suitable for large-scale extraction. Due to high equipment requirements and high economic investment, ultrafiltration was not widely used.^[20]^ In addition, some people have also used immune magnetic beads for the separation of exosomes, which can obtain high-purity exosomes, but it cannot be used in large-scale extraction. Commercial exosomes extract kit^[21]^ is also a quickly method to get exosomes. It is more expensive and only apply in small-scale extraction (Table 1).

**Table 1:** Current methods for isolation of exosomes.

Method	Advantages	Disadvantage
Ultracentrifugation	1. Large-scale extraction 2. Simple operation 3. Conventional and widely used	1. Time-consuming 2. Costly instrumentation
Ultrafiltration	1. High purity 2. Ultracentrifugation is not needed	1. Protein contaminants
Immunomagnetic bead	1. Rapid 2. Simple operation 3. Higher quantities 4. Greater sensitivity	1. Complex and difficult operation 2. Expensive
Commercial exosomes extract kit	1. Ultracentrifugation is not needed 2. Simple operation 3. Rapid	1. Expensive 2. Small-scale extraction

## 4. The composition and biological function of exosomes

Exosomes contain a large number of proteins, nucleic acids, and lipid components closely related to their original. But DNA components were not cargo in exosomes.^[22,23]^ Common proteins in exosomes include proteins related to membrane transport, such as RAB GTPases, annexin, flotillins, MVB producing proteins (ALIX), and tumor susceptibility gene 101 protein. The exosomes also contain 4 transmembrane proteins, including CD9, CD63, and CD81, as well as heat shock protein 60 and heat shock protein 90.^[24–26]^ The exosomes contain various nucleic acids, such as miRNAs and mRNA, which were the first 2 types of RNA^[27,28]^ identified in exosomes, followed by lncRNA, tRNA, snRNA, snoRNA, and circRNA.^[28,29]^ These RNAs have the function of regulator gene expression and can be used as potential biomarkers.^[30]^ Lipid components in exosomes include sphingomyelin, phosphatidylserine, phosphatidylinositol, phosphatidic acid, ceramide, and cholesterol,^[31]^ which can be detected by various methods such as immunoblotting, flow cytometry fluorescence staining, and mass spectrometry analysis. The components cargo in exosomes can be classified into 2 categories: the first category is ordinary proteins, which are related to the biological formation of exosomes and are distributed across all exosomes. For example, ALiX, CD63, TSG101. The second type is specific protein. Allowing to different cell origins, this specific protein is also various,^[32]^ such as diphosphoglycerate, phosphatidylserine, and cholesterol.^[33]^

Due to the wide range of sources of tumor tissue in the human body, the composition of tumor exosomes is more complex. The protein composition of exosomes determines that they have some common functions and can also play unique roles. In general, exosomes have biological functions such as clearing excess cell proteins, intercellular information transmission, and cell membrane repair. Study showed that exosomes can clear excess membrane proteins during the maturation process of red blood cells.^[34]^ Another important function of exosomes is to communicate between cells. After being secreted, exosomes reach the vicinity of the target cell through blood flow. Some protein complexes on their surface are ligands of the target cell, which mediate information transmission between cells through mutual recognition between receptors and ligands.^[35]^ These ligands include CD9 and CD63, which are all 4 transmembrane proteins and are the largest protein family in exosomes. Mittelbrunn et al^[36]^ found that CD63 on exosomes is involved in information transmission between antigen-presenting cells and T lymphocytes. Some studies have also found that 4 transmembrane proteins may participate in the assembly of proteins on exosomes and affect the selection of target cells.^[37]^

## 5. The functional and mechanism of exosomes lncRNA in lung cancer

Lung cancer is a malignant tumor originating from the bronchial mucosa or glands, which is the main cause of cancer-related deaths worldwide. According to the latest GLOBOCAN data released by the International Cancer Agency, in 2020, there are 19,292,789 new cancer patients, of which the incidence rate of lung cancer was 11.4% and ranking second. Meanwhile, lung cancer accounts for 18% of all cancer deaths, ranking first and almost twice the proportion of deaths from colorectal cancer. The incidence rate and mortality of lung cancer rank first^[38]^ especially in male patients. Although early NSCLC can be cured through surgery, more than 50% of NSCLC patients who receive surgical treatment will relapse within 5 years. In addition, due to the lack of highly specific diagnostic biomarkers in clinical practice, up to 70% of patients are diagnosed as advanced and cannot be treated surgically. Therefore, the diagnosis and treatment of lung cancer is one of the main challenges in the field of oncology.

Exosomes are extracellular vesicles with a diameter of 30 to 150 nm secreted by most cells, including tumor cells. They carry and transmit important signaling molecules such as miRNA, long non coding RNA lncRNA, proteins, and mediate material transportation and signal transmission between cells or tissues. In recent years, exosomes miRNAs were proved to participate in tumor metastasis, tumor immune regulation, tumor angiogenesis, and regulation of tumor resistance by controlling the expression of signaling pathway genes, affecting the occurrence and development of lung cancer, providing new ideas for clinical diagnosis and treatment of lung cancer. However, the limited quantity and lack of specificity of exosomes miRNAs limit their widespread application in biomarkers.^[39]^ Evidence suggests that exosomes lncRNA has significant potential in disease diagnosis, occurrence, and development.^[40]^

The length of lncRNA exceeds 200 bp and has various functions such as signal transduction, guiding ribonucleoprotein complexes, and molecular induction. However, due to its lack of protein encoding ability,^[41]^ it has not received sufficient attention for many years. Recently, lncRNA has become a new research hotspot. Many researchers found that lncRNA plays an important role in some physiological conditions, including chronic obstructive pulmonary disease, asthma, and tumor.^[40]^ In lung tumors (Table 2), lncRNA could inhibit miRNA through modifying chromatin remodeling and histone, epigenetic modification or by “sponge” effect, or binding with transcription factors to interfere with the expression of downstream genes and participate in the development process of cancer cells in various ways.^[42]^ Compared to miRNA, lncRNA exhibits better tissue specificity, which make it acts as a new potential biomarker for tumors. In addition, lncRNA also regulate lung tumor angiogenesis and metastasis, drug resistance, and immune function (Table 3).

**Table 2:** Downstream targets of the lncRNAs described in lung tumor.

Lnc RNA	Expression	Signaling pathway	Target genes
HOTAIR	Up regulated	WNT/β-catenin	RB1, E2F1, pULK1
H19	Up regulated	WNT/β-catenin	WNT2, WNT5A, WNT6, WNT10A FOXN1, TCF7
MALAT1	Up regulated	WNT/β-catenin PTEN/PI3K/AKT AKT/MTOR	BCL2, MMP9, PIK3CA, STAT3
CDKN2B-AS1	Down regulated	P53	
GAS5	Down regulated	PTEN/PI3K/AKT	PTEN
TUG1	Down regulated	PTEN/PI3K/AKT	PTEN

**Table 3:** Function of extracellular lncRNA in lung cancer.

Category	Long non-coding RNA	Functions	Action
Angiogenesis and metastasis	Exosomes-lnc-MMP2-2	Migration and invasion	Vimentin, N-cadherin, E-cadherin, MMP2
	Exosomes MALAT-1	Promote cell cycle, reduce cell apoptosis	CyclinD1, cyclinD2, CDK
	Exosome lncRNA UFC1	PTEN, PI3K/Akt	E2F1
	Exosome IncRNA GAS5	Promote proliferation, inhibit apoptosis	miR-29-3p, PTEN PI3K AKT
	Exosomal LINC00662	Proliferation, migration, and invasion	miR-320d
	Exosome FOXD3AS1	Proliferation and invasion	PI3K/Akt ELAVL1
	Exosomal HOTAIR	Proliferation, migration, and invasion	miR-203
Biomarker	Exosome lncRNA GAS5	Tumor suppressor	Lower
	Exosome lncRNA MALAT-1	Tumor promoter	Higher
	Exosome lncRNAs TBILA	Tumor promoter	Higher
	Exosome lncRNAs AGAP2-AS1	Tumor promoter	Higher
Drug resistance	Exosomes lncRNA FOXD3AS1	Inhibit cell apoptosis	5-Fluorouracil
	Exosomes lncRNA UCA1	miR-143/FOSL2 axis	Gefitinib
	Exosomes lncRNA MEG3	miR-15a-5p/CCNE1 axis	Cisplatin
	Exosome lncRNA RP11838N2.4	miR-143	Erlotinib
Immune regulation	Exosomes lncRNA OIP5- AS1	miR-142-5p	Up-regulated
	Exosome lncRNA PCAT-1	miR-182/miR217	Up-regulated

MEG3 = maternal expression gene 3, MMP = matrix metalloproteinase, UCA1 = urothelium cancer associated gene 1.

### 5.1. Extracellular lncRNA participates in angiogenesis and metastasis of lung cancer

Lung cancer cells are the invasiveness and distant metastasis, which are closely related to epithelial mesenchymal transition (EMT). EMT is a phenomenon that occurs under physiological or pathological conditions, where polar epithelial cells undergo transformation into mesenchymal cells with migratory ability. In the early stage of tumor metastasis, tumor cells acquire the characteristics of mesenchymal cells through EMT, migrate across basal cells and vascular endothelial cell layers, thereby promoting tumor metastasis.^[43]^ Transforming growth factor β (TGF-β) is one of the most important EMT inducing factors. It can interact with corresponding membrane receptors, activating downstream proteins of EMT related signaling pathways, and thereby promoting the metastatic of tumor cells.^[44]^ Previous studies showed that TGF-β mediated exosomes miRNAs regulate the migration and invasion of lung cancer cells. In recent years, studies discovered that TGF-β mediated extracellular lncRNA is also closely related to the invasion and metastasis process of tumors. Wu et al^[45]^ found that TGF-β mediated NSCLC derived exosomes lnc-MMP2-2 regulate the migration and invasion of lung cancer cells to blood vessels by up regulating the expression of vimentin and N-cadherin, reducing the expression of E-cadherin, and promoting the expression of matrix metalloproteinase 2. Zhang R. et al^[46]^ found that serum exosomes MALAT-1 can upregulate the expression of cyclinD1, cyclinD2, and CDK in lung cancer patients, promote cell cycle progression, reduce cell apoptosis, and lead to lung cancer cell proliferation and metastasis. The expression of MALAT-1 levels are positively correlated with lung tumor staging and lymph node metastasis.

PI3K/AKT is one of the important molecular pathways in malignant tumors. It widely involved in regulating cell proliferation, differentiation, apoptosis, and migration. According to reports, a tumor suppressor gene with phospholipase activity in macrophages, M2 polarized homolog (PTEN), is involved in regulating PI3K and AKT phosphorylation, playing a key role in lung cancer angiogenesis.^[47]^ Exosome lncRNA UFC1 promoted NSCLC progression through inhibiting PTEN and activating the PI3K/Akt signaling pathway by binding E2F1-mediated epigenetic modification.^[48]^ Cheng et al^[49]^ demonstrated that low-level lung cancer cell derived exosomes growth inhibitory specific gene 5 (IncRNA GAS5) can competitively bind to miR-29-3p, regulate PTEN expression, affect PI3K and AKT phosphorylation in human umbilical vein endothelial cells, promote human umbilical vein endothelial cell proliferation, inhibit apoptosis, and ultimately lead to lung cancer angiogenesis. Exosomal LINC00662 promote lung cancer proliferation, migration, and invasion by binding to miR-320d.^[50]^ Mao et al^[51]^ found that exosomes rich in FOXD3AS1 activate the PI3K/Akt signaling pathway by interacting with ELAVL1, promoting lung cancer cell proliferation and invasion and thereby promoting tumor progression. Exosomal HOTAIR promote lung cancer proliferation, migration, and invasion by sponging miR-203.^[52]^ In addition, GAS5-inficient exosomes original lung cancer cells could promote tumor angiogenesis.^[53]^ MRPL23-AS1-enriched exosomes could promote lung metastasis via acting on pulmonary microvascular endothelial cells.^[54]^

### 5.2. The role of exosomes lncRNA in the diagnosis of lung cancer

Early diagnosis can significantly improve the 5-year survival rate, reduce the risk of metastasis, and prolong survival rate for lung cancer patients, which is closely related to the prognosis of the disease. However, the lack of early diagnostic markers is the main reason for poor prognosis in lung cancer patients. The accumulated evidence suggests that exosomes lncRNAs can serve as promising biomarkers for the lung cancer diagnosis. For example, lncRNA GAS5 is a recently discovered as a tumor suppressor, which involves many cancers, such as breast cancer, prostate cancer, lung cancer, and colorectal cancer. Research found that the expression level of GAS5 in NSCLC patients is lower than that of healthy subjects, which increases the proliferation of tumor cells.^[55]^ Study showed that GAS5 levels are related to TNM staging and tumor size of NSCLC, and serum exosomes GAS5 expression in advanced NSCLC patients is lower than that in early NSCLC patients. The ROC curves showed that the AUC, sensitivity, and specificity of GAS5 in diagnosing NSCLC were 0.857, 85.94%, and 70%. Combined serum exosomes GAS5 with CEA, the AUC increases to 0.929, which showed better detection ability of early NSCLC compared to CEA only (AUC 0.718, sensitivity 50.37%, specificity 85%).^[56]^ Another study showed a significant upregulation of exosomes lncRNA SOX2-OT in lung squamous cell carcinoma (LSCC) patients. In the study, the authors evaluated the diagnostic value of SOX2-OT in distinguishing between LSCC and non LSCC, and reported an AUC of 0.82, with sensitivity and specificity of 0.76 and 0.73. Similarly, the levels of exosomes SOX2-OT were significantly correlated with tumor size, TNM staging, and lymph node metastasis.^[57]^ Zhang R. et al^[46]^ reported that serum exosome lncRNA MALAT-1 was highly expressed in NSCLC patients. The ROC curve showed that detecting serum exosomes MALAT-1 as a diagnostic biomarker of NSCLC, its AUC, sensitivity, and specificity were 0.703, 0.601, and 0 809. In addition, exosomal lncRNAs, such as DLX6-AS1^[58]^ and RP11-510M2^[59]^ can be used as blood noninvasive diagnostic biomarkers of lung cancer. The exosomal lncRNAs TBILA and AGAP2-AS1 were found up-regulated in NSCLC. Combination of these 2 lncRNAs and Cyfra21-1 improved the NSCLC diagnostic accuracy by evaluating by ROC curve.^[60]^

Actually, single miRNAs or exosomal miRNAs were also suggested as clinical diagnosis biomarkers of lung cancer. However, the differences in their expression levels between healthy people and patients are usually quite tiny. In addition, only several miRNAs were differently expression in plasma exosomes. For example, plasma levels of miR-19b-3p, miR-21-5p, miR-221-3p, miR-409-3p, miR-425-5p, and miR-584-5p were elevated in lung adenocarcinoma patients, but only miR-19-3p, miR-21-5p, and miR-221-3p were found to be upregulated in plasma exosomes.^[61]^ This observation implied the importance of selecting a proper sampling method for certain circulating miRNAs. Therefore, exosomes lncRNAs can serve as promising biomarkers for the lung cancer diagnosis.

### 5.3. Exosome lncRNA regulates drug resistance in lung cancer

LncRNA plays various types of regulating functions in cellular processes, including interacting with DNA, RNA, or proteins to form complexes and controlling the expression of target genes. lncRNA plays a role in cancer development and is closely related to metastasis, recurrence, prognosis, and chemotherapy resistance through various mechanisms. LncRNA can enter exosomes through RNA binding proteins and perform biological functions after uptaken by target cells. The heterogeneous nuclear ribonucleoprotein hnRNPA2B1 is an RNA binding protein that plays an important role in many biological processes such as mRNA maturation, transport and metabolism, and gene regulation of long noncoding RNA. HnRNPA2B1 can bind to the 5′ end specific sequence (GGAG) of lncRNA and participate in lncRNA embedding into extracellular vesicles.^[62]^ Current research shows that lncRNA AGAP2-AS1, lncRNA LNMAT2, and lncRNA H19 enter the exosomes through binding with hnRNPA2B1 at the 5′ end sequence.^[63]^ At present, the tumor chemoresistance mediated by exosome lncRNA is mainly seen in cytotoxic drugs like microRNA, such as cisplatin, oxaliplatin, temozolomide acting on the chemical structure of DNA, 5-fluorouracil (5-FU) affecting nucleic acid formation, and doxorubicin acting on nucleic acid transcription.

For lung cancer patients who have lost the opportunity for surgery in the late stage, radiotherapy, chemotherapy, and targeted therapy have brought new hope, which have become a new direction of precision medicine for NSCLC. However, almost all NSCLC patients who receive targeted drug treatment inevitably experience acquired drug resistance. More and more evidence showed that exosomes lncRNA participates in acquired drug resistance in lung cancer cells by regulating gene expression of related signaling pathways and interacting with miRNAs. Study showed that,^[64]^ sensitive tumor cells uptake exosomes lncRNA H19 can lead to resistance of these receptor cells to erlotinib and gefitinib, respectively. Mao et al^[51]^ showed that exosomes lncRNA FOXD3AS1 can inhibit cell apoptosis induced by 5-FU and increase cancer cell resistance to 5-FU. They showed that the effects of FOXD3-AS1 rich exosomes from lung cancer cells on proliferation, invasion, and 5-FU resistance. Online bioinformatics database analysis shows that FOXD3-AS1 is upregulated in lung cancer progression. The RT-qPCR confirmed that FOXD3-AS1 was upregulated in lung cancer tissues and cell lines, and FOXD3-AS1 was enriched in exosomes derived from lung cancer cells. In addition, FOXD3-AS1 and ELAVL1 proteins can bind to each other and upregulate their expression. Exosomes derived from lung cancer cells can promote the proliferation and invasion of A549 cells, inhibit 5-FU induced apoptosis, and transfection of si-FOXD3-AS1 or si-ELAVL1 into A549 cells cultured with exosomes can reverse these effects. Exosomes derived from lung cancer cells activate the PI3K/Akt pathway, transfect si-FOXD3-AS1, or using the PI3K inhibitor LY294002 could reverse activation of the PI3K/Akt axis induced by exosomes. So FOXD3-AS1 in exosomes derived from lung cancer cells can upregulate the expression of ELAVL1 and activate the PI3K/Akt pathway to promote lung cancer progression and resistance to 5-FU, providing a new strategy for the treatment of lung cancer. Chen et al^[65]^ observed the presence of the UCA1/miR-143/FOSL2 axis in gefitinib resistant in NSCLC cells. The exosome lncRNA urothelium cancer associated gene 1 was significantly up-regulated in gefitinib resistant NSCLC cell lines, and promoted the resistance of NSCLC cells to gefitinib through the miR-143/FOSL2 axis. Knocking out UCA1 can promote the expression of miRNA-143, which plays a role in reversing gefitinib resistance by regulating the expression of FOSL2. Sun et al^[66]^ found that overexpression of cancer cell associated fibroblast derived exosomes lncRNA maternal expression gene 3 in small cell lung cancer tissue promotes the metastasis of SCLC cells and enhances the resistance of SCLC to cisplatin through the miR-15a-5p/CCNE1 axis.

Currently, research on the acquired resistance of extracellular lncRNAs in NSCLC is more focused on targeted drugs such as EGFR-TKIs. Lei et al^[64]^ showed that the RNA binding protein hnRNPA2B1 interacts with lncRNA H19 and is responsible for packaging H19 into exosomes and transporting it to non-resistant cells, thereby inducing resistance to gefitinib. Pan et al^[67]^ found that exosomes H19 mediate NSCLC resistance to erlotinib through the miR-615-3p/autophagy related gene (ATG) 7 axis. For third-generation EGFR-TKI oxitinib, compared with sensitive plasma, H1975 cells, and extracellular vesicles, the relative expression levels of lncRNA-MSTRG.292666.16 in oxitinib resistant plasma, H1975R cells, and their extracellular vesicles were significantly upregulated. Knocking out lncRNAMSTRG.292666.16 can reduce the resistance of H1975R cells to oxitinib, and the resistant exosomes transmit resistance in sensitive H1975 cells, The study also confirmed that exosomes resistant to oxitinib can regulate miRNA-21, miRNA-125b, and TGF induced by oxitinib-β and some target genes such as ARF6 and c-Kit.^[68]^

Macrophages play an important role in the drug resistance of cancer cells. They are a heterogeneous immune cell group and one of the main groups of tumor infiltrating immune cells, which can differentiate into 2 different types, including M1 macrophages and M2 macrophages. In the tumor microenvironment, macrophages display M2 phenotype, which is defined as tumor associated macrophages. Tumor associated macrophages are the main participants and coordinators of tumor progression in the microenvironment. Research has shown that a high M2/M1 macrophage ratio contributes to the proliferation, metastasis, invasion, and drug resistance of cancer cells. Zhou et al^[69]^ found that NSCLC cells target and regulate the miR-627 3p/Smads signaling pathway, transferring cancer cell derived exosomes lncRNA SOX2 transcripts (SOX2-OT) to macrophages, thereby promoting macrophage M2 polarization, causing M2/M1 macrophage ratio imbalance, and enhancing their own EGFR-TKIs resistance. Deng et al^[68]^ showed that M2XI type TAM derived exosomes lncRNA MSTRG 292666.16 may control miR-6836-5p/MAPK8IP3 axis to promote the resistance of NSCLC cells to osetinib and plays a key role in the spread of drug resistance in tumor cells. Zhang F. et al^[70]^ found an increase in the expression of M2 macrophage derived exosomes (AGAP2-AS1) in lung cancer tissues that are resistant to radiation therapy by downregulating miR-296 and overexpressing NOTCH2. Recent studies^[71]^ showed that exosomes lncRNA SNHG7 also induces resistance to docetaxel in lung adenocarcinoma cells through multiple pathways. Firstly, the exosomes IncRNA SNHG7 induce autophagy in lung adenocarcinoma cells by recruiting human antigen R to stabilize ATG5 and ATG12; secondly, exosomes SNHG7 triggers macrophage M2 polarization by recruiting Cullin4A, promotes PTEN ubiquitination, and activates the PI3K/AKT pathway. By inducing autophagy in lung adenocarcinoma cells and promoting macrophage M2 polarization, it promotes the resistance of lung adenocarcinoma cells to docetaxel. Finally, lung adenocarcinoma cells resistant to docetaxel can promote the resistance of parental lung adenocarcinoma cells to docetaxel by secreting the exosomes SNHG7.

### 5.4. Extracellular IncRNA participates in immune regulation of lung cancer

Tumor microenvironment (TME) is a highly dynamic complex environment composed of fibroblasts, immune cells, endothelial cells, stromal cells, and extracellular matrix. During the occurrence and development of tumors, the interaction between TME and tumor cells mediates the immune tolerance of tumor cells, affecting the clinical efficacy of immunotherapy. Research shows that exosomes lncRNA can regulate the occurrence and development of lung cancer by influencing the function of immune cells in the TME, and mediating the immune escape of tumor cells. Jiang et al^[9]^ found that the exosomes lnc RNA OIP5-AS1 original CAF, weakens the inhibitory effect of miR-142-5p on PD-L1 by inhibiting the expression of miR-142-5p, upregulates PD-L1 expression, and inhibits the cytotoxicity of lung cancer cells induced by mononuclear cells. Recent studies showed that^[72]^ PD-1 interacts with the PD-L1 receptor on immune cells, leading to T lymphocyte apoptosis and immune escape. In addition, the study^[73]^ found that in tumor tissues with metastasis or KRAS mutation, the expression of exosome lncRNA PCAT-1 was significantly up-regulated, which regulated KRAS related chemotherapy tolerance through miR-182/miR217 and closely related to the reduced survival rate and poor prognosis of lung cancer patients. Down regulation of PCAT-1 can induce T cell recruitment and activate IL-13/IL-33 mediated pathway, inhibit tumor metastasis caused by CAF mediated activation of stromal fibroblasts, enhance the sensitivity of tumor cells to chemotherapy drugs and affect the microenvironment of tumor immune cells.

## 6. Conclusion

In recent years, exosome lncRNAs were proved to serve as regulatory factors for gene expression, mediating intercellular communication, and participating in the occurrence and development of various diseases. The impact of exosomes on cell biology is greater than originally expected, which makes related research rather complex. The temporal and spatial expression patterns, precise roles and mechanisms of specific lncRNAs encapsulated in exosomes remain largely unknown in different system development and diseases. Currently, exosomal lncRNAs are involved in pathological cellular processes, promote cell-to-cell and tissue-to-tissue crosstalk, modulate autoimmune diseases, cardiovascular diseases and tumors. However, much effort remains to be done to understand the full extent of exosomal ncRNAs exerting their pathological effects.^[74]^ Actually, exosomes lnc RNA has advantages on the early diagnosis of lung cancer, tumor cell metastasis, drug resistance, and immune regulation. Research on the prognosis of lung cancer and the development of drug resistant sensitizers for drug-resistant patients is also ongoing. The various research findings can provide some unique ideas on how to improve the efficiency of diagnosis and treatment of lung cancer in the future. Further research is needed to elucidate the relevant mechanisms of action such as lncRNA and miRNA in tumors, in order to create conditions for early and targeted clinical interventions for tumor patients, and to address the issues of radiochemotherapy resistance and other cancer related treatments during patient treatment.

## 7. Limitations to the overview

The current research progress on the role of lncRNAs in lung cancer is limited to basic research, and there are currently almost no relevant research reports indicating that these research results have been applied to new drug discovery or clinical treatment in clinical trials. In addition, we need to determine the extent to which purified exosomes are likely to be sufficient to confer the positive effects. The dynamics and pharmacokinetics, as well as toxic studies of potential exosomal lncRNAs drugs require to be repeatedly tested. Further experiments in animal are necessary to evaluate the pathological functions of exosomal lncRNAs.

## Acknowledgments

The authors thank the reviewers for their valuable comments on this study.

## Author contributions

**Conceptualization:** Zhenan Xu.

**Data curation:** Jingyuan Jiang.

**Formal analysis:** Fengwu Lin.

**Funding acquisition:** Wenqi Wu.

**Methodology:** Zhe Zhang.

**Software:** Chen Zhang.

**Validation:** Dongliang Qin.
